# TRIM27 promotes the development of esophagus cancer via regulating PTEN/AKT signaling pathway

**DOI:** 10.1186/s12935-019-0998-4

**Published:** 2019-11-08

**Authors:** Liang Ma, Ninghua Yao, Ping Chen, Zhixiang Zhuang

**Affiliations:** 1 Department of Oncology, The Second Affiliated Hospital of Soochow University, Sanxiang Road No. 1055, Gusu District, Suzhou, 215004 Jiangsu China; 2 Department of Oncology, First People’s Hospital of Yancheng, The Forth Affiliated Hospital of Nantong University, Yulong West Road No.166, Tinghu District, Yancheng, 224001 Jiangsu China; 3 Departments of Radiotherapy, Affiliated Hospital of Nantong University, Nantong, Jiangsu China

**Keywords:** Esophagus cancer, TRIM27, PI3/AKT, PTEN

## Abstract

**Background:**

Tripartite motif‑containing 27 (TRIM27) belongs to the TRIM protein family, which is closely related to the progression of some certain human cancers. Nevertheless, the biological function of TRIM27 in esophageal squamous cell carcinoma (ESCC) is still not clear. The aim of present research is to examine the function of TRIM27 in ESCC cells.

**Methods:**

In the present study, RNA interference (RNAi) and lentiviral vector were used to knockdown and overexpression of TRIM27 in ESCC cells respectively. qRT-PCR and western blot were used to examine the expression of TRIM27 in ESCC cells. Cell counting kit-8 (CCK-8) assay was performed to determine the proliferation of cells.

**Results:**

Our analyses indicated that TRIM27 was a pro-proliferation factor in ESCC cells. Moreover, overexpression of TRIM27 deeply suppressed the apoptosis of ESCC cells and accelerated its glucose uptake. In addition, an AKT inhibitor LY294002 was used to determine the connection between TRIM27 and AKT in ESCC cells. Our results demonstrated that TRIM27 has involved in the PI3/AKT signaling pathway. Moreover, TRIM27 interacted with PTEN and mediated its poly-ubiquitination in ESCC cells. Importantly, the glycolysis inhibitor 3-BrPA also inhibited the effect of TRIM27 on ESCC cells. Hence, TRIM27 also participated in the regulation of energy metabolism in ESCC cells.

**Conclusions:**

This research not only gained a deep insight into the biological function of TRIM27 but also elucidated its potential target and signaling pathway in human ESCC cells.

## Background

Esophageal squamous cell carcinoma (ESCC) is one of the common death related cancers worldwide, which ranks the sixth place in cancer mortality [1]. Although the traditional treatments (radiation, chemotherapy or esophagogastric resection) contribute to the treatment of ESCC patients, the overall 5-year survival rate is still less than 20% [2]. Previous report has demonstrated that ESCC can be possibly cured at its early phase [3]. Therefore, the effective biomarkers for diagnosing ESCC are urgently needed.

Tripartite motif‑containing 27 (TRIM27) is one of the family members of TRIM, which inherits the basic structure of TRIM family [4]. As a DNA binding protein, TRIM27 consists of a RING finger protein domain, a B‑box type I domain, and a B‑box type II domain and exhibits a RBCC motif at the N-terminus [5]. Previous report has demonstrated that TRIM27 accelerates the progression of colorectal cancer [5]. Moreover, it have been confirmed that inhibiting the expression of TRIM27 suppresses the proliferation of ovarian and nasopharyngeal cancer cells respectively [6, 7]. In addition, TRIM27 positively regulates the TNF-α-induced apoptosis [8]. Importantly, some single nucleotide polymorphisms (SNPs) of TRIM27 is associated with human ESCC [9]. However, the underlying molecule network of TRIM27 is still less identified in human ESCC.

PI3K/AKT signaling pathway plays a key role in the progression of human cancers, which is identified as a promising target for anti-cancer therapy [10]. Previous reports have confirmed that TRIM59 and TRIM27 promote the proliferation of CRC cells through regulating PI3K/AKT signaling pathway [5, 11]. Moreover, TRIM14 promotes the activity of PI3K/AKT signaling pathway in osteosarcoma cells [12]. Further, suppressing the PI3K/AKT signaling pathway contributes to inhibiting the proliferation of ESCC cells [13, 14]. However, the detailed relationship between TRIM27 and PI3/AKT pathway remains unclear in human ESCC cells.

The aim of present study is to explore the biological function of TRIM27 in ESCC cells. RNA interference (RNAi) and lentiviral vector were used to silencing and overexpression of TRIM27 in human ESCC cells. Our research not only provided novel evidences to understand the biological function of TRIM27 in human ESCC cells but also elucidated its potential target and signaling pathway in ESCC cells.

## Materials and methods

### ESCC tissue specimens

Esophageal squamous cell carcinoma tissues (n = 25) and adjacent-matched noncancerous samples (n = 18) were collected from The Second Affiliated Hospital of Soochow University, Suzhou, Jiangsu, China. All tissues were snap-frozen in liquid nitrogen, stored at − 80 °C.

### Cell culture

The cell lines used in this research were TE-1, TE-11, ECA-109, KYSE150 and HEEC, which were purchased from Shanghai biology institute (Shanghai, China). 10% fetal bovine serum (GIBCO, USA) was added to culture media that containing 2 mM l-glutamine and 1% penicillin/streptomycin (Solarbio, China). Cells were grown in DMEM Medium (Trueline, USA) and maintained in a 5% CO_2_, at 37 °C incubator. This study was in agreement with the Declaration of Helsinki. The AKT inhibitor LY294002 (25 μmol/L, S1105, Selleck, USA) and glycolytic inhibitor 3-BrPA (20 μmol/L, 1113-59-3, Sigma, USA) were dissolved in DMSO (D2650, Sigma, USA) and used to culture cells.

### RNA isolation and real-time PCR

Total RNA from different samples were isolated by using TRIzol Reagent (Invitrogen, USA). Then, cDNA synthesis kit (Fermentas, Canada) was used to reverse transcribe RNA into complementary DNA (cDNA). The program of the real-time PCR reaction was listed as follows: 95 °C for 10 min followed by 40 cycles of 95 °C for 15 s and 60 °C for 45 s. GAPDH was used to normalize the gene expression. 2^−ΔΔ*Ct*^ method was applied to calculate the relative gene expression. All data represented the mean of three replicates. Primer sequences are provided in Additional file 1.

### Knockdown and overexpression of TRIM27

For silencing human TRIM27 (NM_006510.4), three shot interference RNAs (siRNA) that targeting TRIM27 were synthesized (Major, Shanghai, China) and subsequently transfected into the KYSE150 and TE-11 cells respectively by using Lipofectamine 2000 (Invitrogen, USA). Meanwhile, a nonspecific scrambled siRNA sequence was transfected into KYSE150 and TE-11 cells as negative control (siNC). The targeting locus and sequence of TRIM27 siRNA is provided in Additional file 1: Table S1.

As for overexpression of TRIM27, a lentiviral plasmid (pLVX-puro) containing the full-length human TRIM27 cDNA sequence and a mock plasmid (oeNC) were transiently transfected into TE-1 cells respectively.

### Western blot

RIPA lysis buffer (JRDUN, Shanghai, China) was used to extract protein as indicated. An enhanced BCA protein assay kit (Thermo Fisher, USA) was utilized to estimate the protein content. Total protein (25 μg) was fractionated by using 10% SDS-PAGE and transferred to a nitrocellulose membrane (Millipore, USA) for 2 h, which were probed at 4 °C for 12 h with the primary antibodies followed by incubation for 1 h at 37 °C with the secondary antibody (HRP-labeled goat anti rabbit IgG antibody; 1:1000, Beyotime, China). An enhanced chemiluminescence system (Tanon, China) was utilized to quantify the protein content. Each analysis was detected in triplicate. GAPDH was used as the internal reference. The crucial information of the primary antibodies was listed in Additional file 1: Table S2.

### Cell proliferation assay

Cell counting kit-8 (CCK-8) assay kits (SAB, USA) was used to examine cell proliferation. All procedures were performed according to the instruction of the manufacture. In brief, cells were seeded in 96-well plates and incubated with CCK-8 solution (1:10) for 1 h. Then, OD450 value was examined by microplate reader (Pulangxin, China) at 12, 24 and 48 h after seeding. The experiment was independently repeated thrice at each time point.

### Cell apoptosis

Briefly, Annexin V-fluorescein isothiocyanate (FITC) apoptosis detection kit (Beyotime, China) was used to examine the apoptosis rate of cells according to the instructions of the manufacturer. After 48 h of viral infection, flow cytometer (BD, USA) were utilized to determine cells. Three replications were needed for each sample.

### Glucose transport

In brief, the glucose analog 2-NBDG kits (Biovision, San Francisco, USA) were used as a fluorescent probe for determining the activity of Glucose transport. In order to examine the uptake of 2-NBDG, a total of 5 × 10^5^ cells from different groups were seeded in 6-well plates. Then, all cells were pre-incubated in Krebs–Ringer bicarbonate (KRB) buffer (glucose free) for 15 min after maintaining in a 5% CO_2_ atmosphere at 37 °C for 24 h. After that, cells were incubated in fresh KRB buffer supplemented with 2-NBDG for 45 min at 37 °C, 5% CO_2_. Flow cytometry using a GloMax^®^-Multi + flow cytometer (Promega, USA) was used to quantitatively analyze the stained cells.

### Co-immunoprecipitation (Co-IP)

For IP, whole-cell extracts were prepared after transfection or stimulation with appropriate ligands, followed by incubation overnight with the appropriate antibodies plus Protein A/G beads (Santa Cruz Biotechnology, USA). Beads were washed five times and separated by SDS-PAGE. Western blot was performed by using the antibodies as indicated above.

### Ubiquitination assay

KYSE150 cells were transfected with siNC or siTRIM27, the cells were lysed in 1% SDS-containing radio immunoprecipitation assay (RIPA) buffer by sonication on ice. Then, Lysates were treated by Protein A/G PLUS-Agarose (Santa Cruz Biotechnology, USA) for 1 h. After that, each samples were incubated with the IgG (Proteintech, USA) overnight at 4 °C. Then, the nuclear pellet was collected by centrifugation at 3000 rpm for 5 min at 4 °C and subsequently washed by Protein A/G Plus-Agarose beads for four times. The purified proteins were separated by 4–20% gradient SDS-PAGE. Anti-TRIM27 antibody (12205-1-AP, Proteintech, USA), Anti-PTEN (ab32199, Abcam, UK) and anti-Ubiquitin antibody (ab7780, Abcam, UK) antibody were used for immunoblotting.

### Statistical analysis

Statistical analyses were performed by using the software of GraphPad Prism Version 7.0 (CA, USA). Data were presented by mean ± SD. ANOVA for multiple comparisons was used to determine statistical significance and *p*-value < 0.05 was accepted.

## Results

### TRIM27 was upregulated in ESCC tissues and cells

**Fig. 1:**
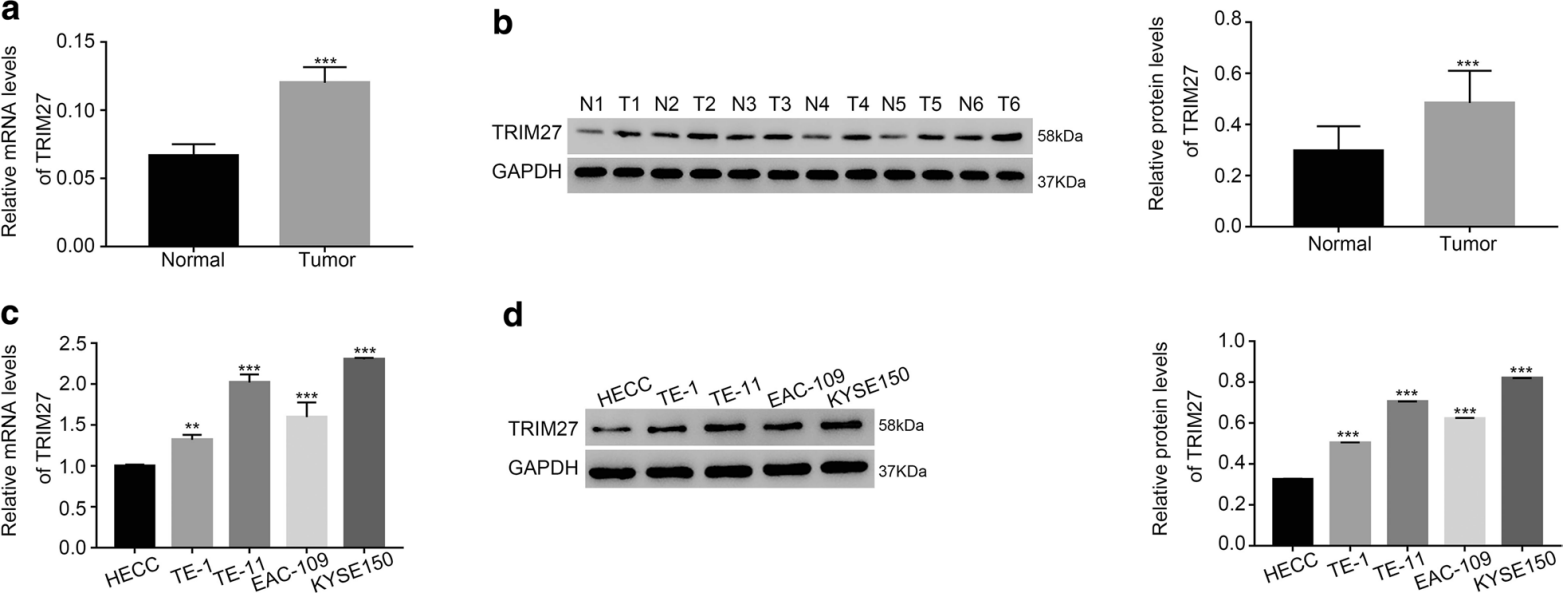
TRIM27 was upregulated in ESCC tissues and cells. *N* normal, *T* tumor. **a** The relative mRNA level of TRIM27 detected in ESCC tissues (n = 25) and adjacent noncancerous samples (n = 18), ***p < 0.001 vs normal. **b** The protein content of TRIM27 examined in six pairs of ESCC tissues and matched adjacent samples, ***p < 0.001 vs normal. **c**, **d** Stands for the mRNA and protein level of TRIM27 in HECC, TE-1, TE-11, EAC-109 and KYSE150 cells respectively, ***p < 0.001 vs HECC

A total of 25 ESCC tissues and 18 adjacent-matched noncancerous samples were used to examine the mRNA level of TRIM27. Moreover, six pairs of ESCC tissues and matched noncancerous samples were utilized to quantify the protein content of TRIM27. Both the mRNA and protein level of TRIM27 were significantly increased in ESCC tissues compared with that of adjacent samples (Fig. 1a, b). Therefore, our results demonstrated that TRIM27 was upregulated in ESCC tissues.

Moreover, qRT-PCR and western blot were used to determine the mRNA and protein level of TRIM27 in four ESCC cell lines, including TE-1, TE-11, EAC-109 and KYSE150. The esophageal epithelial cells HECC were used as control. In the present study, the level of TRIM27 was also upregulated in ESCC cells compared with that of HECC, especially in TE-11 and KYSE150 cells (Fig. 1c, d). Therefore, knockdown of TRIM27 was induced in TE-11 and KYSE150 cells respectively. Meanwhile, TE-1 cells were chosen for overexpression of TRIM27.

### Knockdown and overexpression of TRIM27 in ESCC cells

**Fig. 2:**
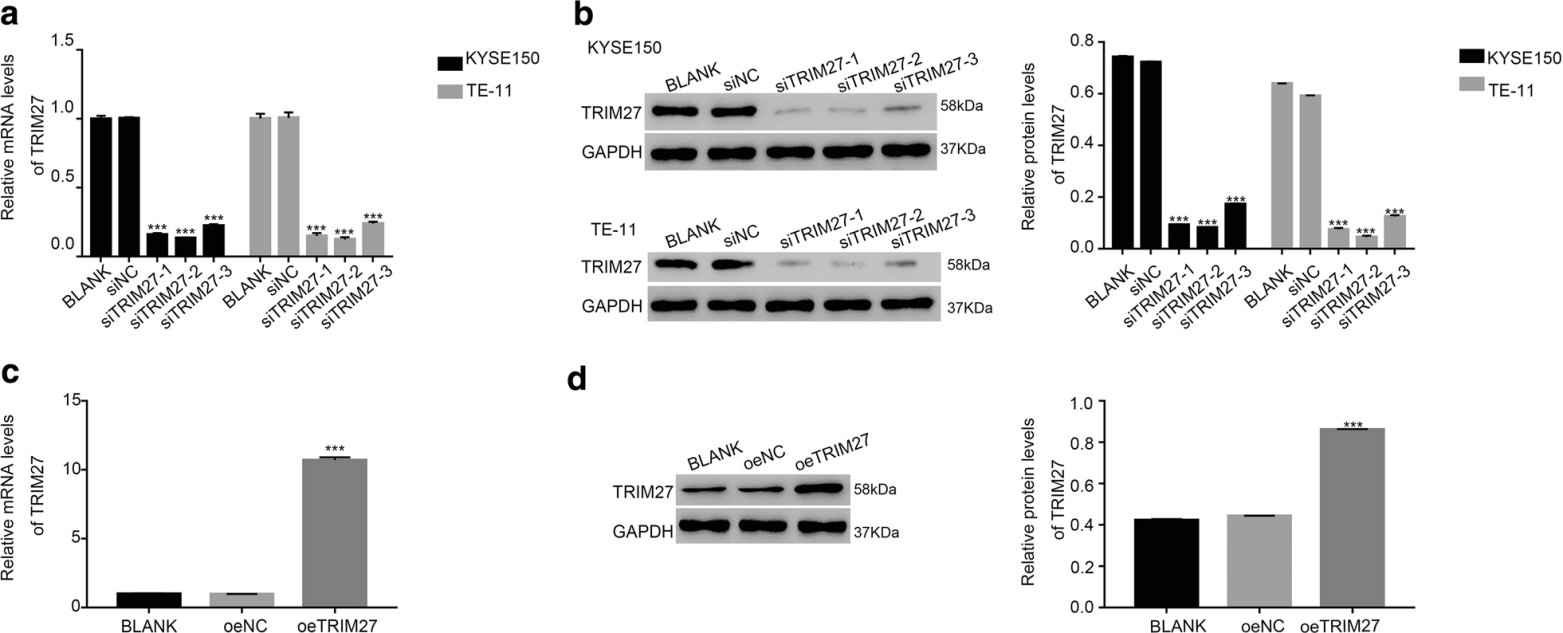
Knockdown and overexpression of TRIM27 in ESCC cells. **a**, **b** The mRNA and protein level of TRIM27 was deeply suppressed by siTRIM27 in KYSE150 and TE-11 cells, ***p < 0.001 vs siNC. **c**, **d** The TRIM27 was significantly upregulated by oeTRIM27 in TE-1 cells. ***p < 0.001 vs oeNC

To further examine the function of TRIM27 in ESCC cells, we synthesized three short interference RNAs (siRNAs) targeting human TRIM27 (siTRIM27-1, siTRIM27-2 and siTRIM27-3) and a nonspecific scrambled siRNA (siNC) and subsequently transfected into KYSE150 and TR-11 cells. The unprocessed cells were served as blank control (BLANK). All TRIM27-siRNAs significantly suppressed the expression of endogenous TRIM27. Cells transfected with siTRIM27-1 and siTRIM27-2 showed a stronger effect than that of siTRIM27-3 in ESCC cells. Therefore, siTRIM27-1 and siTRIM27-2 transfected cells were used for the following analyses (Fig. 2a, b).

Meanwhile, TE-1 cells were transfected with a plasmid for overexpressing TRIM27 (oeTRIM27). Both the mRNA and protein content of TRIM27 was significantly overexpressed in oeTRIM27-transfected cells (Fig. 2c, d). Therefore, the oeTRIM27 transfected cells were utilized for the following analyses.

### Knockdown of TRIM27 suppressed the growth of ESCC cells

**Fig. 3:**
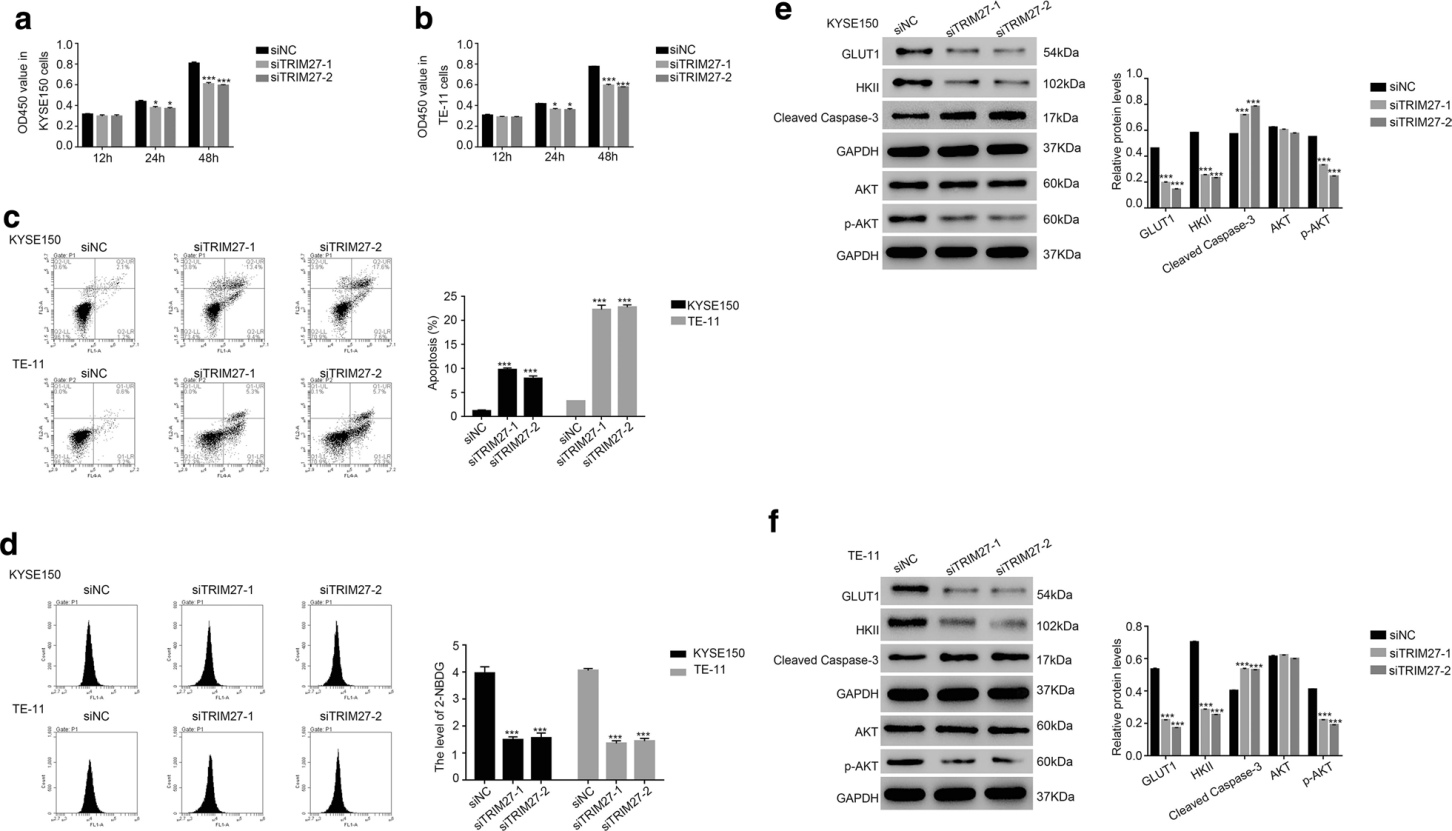
Knockdown of TRIM27 inhibited the growth of ESCC cells. **a**, **b** The cell proliferation rate detected at 12 h, 24 h and 48 h after transfection with siNC, siTRIM27-1 and siTRIM27-2 in KYSE150 and TE-11 cells respectively, *p < 0.05 vs siNC, ***p < 0.001 vs siNC. **c** Cell apoptosis profile was upregulated in siTRIM27 transfected cells as indicated, ***p < 0.001 vs siNC. **d** The level of 2-NBDG was suppressed in siTRIM27 transfected cells, ***p < 0.001 vs siNC. **e**, **f** The protein level of GLUT1, HKII, cleaved caspase-3, AKT and p-AKT examined in different cells as indicated, ***p < 0.001 vs siNC

Cell proliferation rate was examined by cell counting kit-8 (CCK-8). As shown in Fig. 3a, b, siTRIM27-1 and siTRIM27-2 were deeply suppressed the proliferation rate of ESCC cells in two cell lines. Therefore, TRIM27 presented the pro-proliferation property in ESCC cells. Moreover, we also analyzed the apoptosis profile of siTRIM27 transfected cells. Obviously, the apoptosis profile of siTRIM27-1 or siTRIM27-2 transfected cells was much higher than that of siNC transfected cells (Fig. 3c). These results elucidated the anti-apoptosis function of TRIM27 in ESCC cells.

### Glucose transport activity was deeply suppressed by siTRIM27 in ESCC cells

The abnormal of energy metabolism is one of the biochemical characters for cancer cells, which leads to the acceleration of glycolysis activity [15]. In this study, we analyzed the activity of glycolysis by using the glucose analog 2-NBDG. Interestingly, the level of 2-NBDG was deeply suppressed in ESCC cells transfected with siTRIM27-1 or siTRIM27-2 (Fig. 3d). Therefore, TRIM27 might promote the proliferation of human ESCC cells through enhancing the glycolysis activity.

Previous reports have demonstrated that GLUT1 and HKII are positive correlative with the activity of glycolysis [16–19]. To further examine the function of TRIM27 in glycolysis, we quantified the protein content of GLUT1 and HKII in different cells as indicated. The protein content of GLUT1 and HKII were significantly decreased in siTRIM27 transfected cells (Fig. 3e, f). Moreover, the protein level of cleaved caspased-3 was remarkably upregulated in siTRIM27-1 or siTRIM27-2 transfected cells. In addition, the phosphorylation of AKT (p-AKT) was also inhibited in siTRIM27-1 or siTRIM27-2 transfected cells.

### The AKT inhibitor LY29400 and glycolytic inhibitor 3-BrPA abolished the function of TRIM27 in ESCC cells

In order to further explore the correlation between TRIM27 and AKT, the AKT inhibitor LY294002 (25 μmol/L) was used to culture cells transfected with oeTRIM27. Meanwhile, the oeTRIM27 transfected cells were also cultured in the present of the glycolytic inhibitor 3-BrPA (20 μmol/L).

**Fig. 4:**
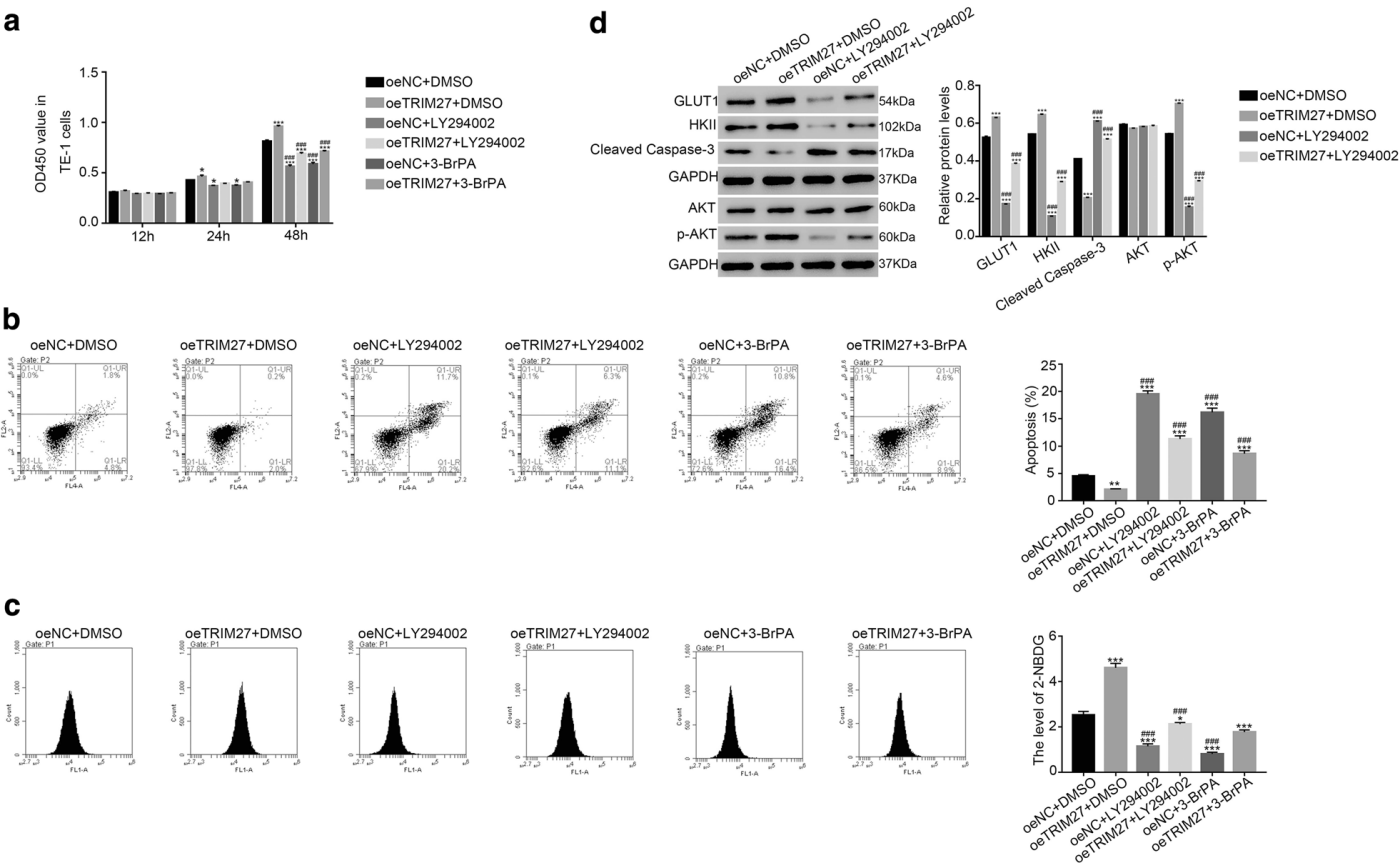
The inhibitor LY294002 and 3-BrPA inhibited the effect of TRIM27 in ESCC cells. **a** The cell proliferation rate detected at 12 h, 24 h and 48 h examined in different cells as indicated. *p < 0.05 vs oeNC, ***p < 0.001 vs oeNC. **b** Cell apoptosis profile was suppressed in oeTRIM27 transfected cells. **p < 0.01 vs oeNC, ***p < 0.001 vs oeNC; ^###^*p *< 0.001 vs oeTRIM27. **c** The level of 2-NBDG was upregulated in oeTRIM27 transfected cells. *p < 0.05 vs oeNC, ***p < 0.001 vs oeNC; ^###^*p *< 0.001 vs oeTRIM27. **d** The protein level of GLUT1, HKII, cleaved caspase-3, AKT and p-AKT examined in different cells as indicated, ***p < 0.001 vs oeNC; ^###^*p *< 0.001 vs oeTRIM27

The cell proliferation rate was significantly promoted in oeTRIM27 transfected cells (Fig. 4a). However, the AKT inhibitor LY294002 deeply suppressed the cell proliferation rate of oeNC and oeTRIM27 transfected cells. Meanwhile, the similar result was also obtained in 3-BrPA cultured cells. Moreover, the apoptosis rate was deeply inhibited in oeTRIM27 transfected cells. Interestingly, the inhibitor LY294002 and 3-BrPA remarkably promoted the cell apoptosis rate in oeNC or oeTRIM27 transfected cells (Fig. 4b).

In addition, we also analyzed the activity of glucose transport in different cells as indicated. It was clearly identified that overexpression of TRIM27 promoted the transport of glucose in cells transfected with oeTRIM27. Moreover, the inhibitor LY294002 and 3-BrPA deeply inhibited the glucose transportation in oeTRIM27 transfected cells (Fig. 4c). As shown in Fig. 4d, the protein content of GLUT1 and HKII were significantly increased in oeTRIM27 transfected cells. However, the AKT inhibitor LY294002 deeply inhibited the expression of GLUT1 and HKII in oeNC or oeTRIM27 transfected cells. Moreover, the protein level of cleaved caspase-3 showed the opposite results as that of GLUT1 and HKII in oeTRIM27 transfected cells. Importantly, the phosphorylation of AKT was positively correlated with TRIM27, which was deeply suppressed by the inhibitor LY294002 and 3-BrPA. Taken together, all these results demonstrated that the effect of TRIM27 was deeply abolished by the AKT inhibitor LY294002 and 3-BrPA on ESCC cells.

### TRIM27 interacted with PTEN and promoted its poly-ubiquitination in ESCC cells

**Fig. 5:**
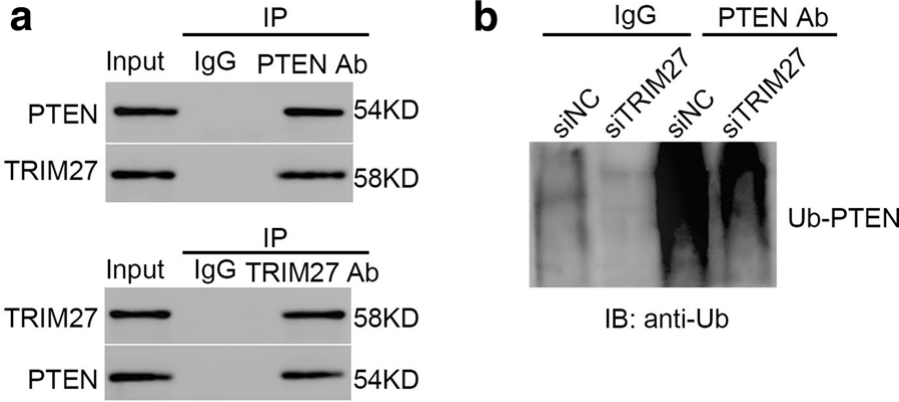
TRIM27 interacted with PTEN and promoted its poly-ubiquitination in ESCC cells. **a** CoIP assay was performed to examine the interaction between TRIM27 and PTEN in ESCC cells. **b** siTRIM27 inhibited the poly-ubiquitination of PTEN in ESCC cells

PTEN is identified as a critical component in PI3/AKT pathway [20]. PTEN/AKT pathway is the common abnormal signaling pathway in human cancers [21]. In order to further examine the function of TRIM27 in PI3/AKT, we analyzed the interaction between TRIM27 and PTEN by co-immunoprecipitation (Co-IP). As shown in Fig. 5a, it was easily identified that there was a stronger interaction between TRIM27 and PTEN. These results indicated that TRIM27 directly interacted with PTEN in ESCC cells.

Previous report has demonstrated that the ubiquitination of PTEN is critical for its stability and nuclear localization [22]. Enhancing the ubiquitination of PTEN has improved the activity of PI3/AKT pathway [23]. In this study, intracellular ubiquitination assays was established to examine whether TRIM27 affected the poly-ubiquitination of PTEN. As shown in Fig. 5b, knockdown of TRIM27 deeply reduced the level of PTEN poly-ubiquitination in ESCC cells. Therefore, TRIM27 not only interacted with PTEN but also promoted its poly-ubiquitination in ESCC cells.

## Discussion

Esophageal squamous cell carcinoma is one of the common tumors of digestive tract. Numerous deaths are caused by ESCC all over the world [24]. Worse still, the outcome of traditional therapy is far from satisfactory. Therefore, enhancing the understanding into the molecule pathogeny of ESCC is a critical step for developing novel strategies for its treatment.

At present study, we systemically explored the function of TRIM27 in ESCC cells. Knockdown and overexpression of TRIM27 were induced in ESCC cells respectively. Analyses from those two experimental sections were consistent. Therefore, our conclusions were more credible.

TRIM protein family are closely related to the development of human tumors [25]. In this study, we found TRIM27 was upregulated in ESCC tissue and cells. Moreover, our results indicated that TRIM27 was a pro-proliferation factor in ESCC cells. Meanwhile, overexpression of TRIM27 deeply inhibited the apoptosis of ESCC cells. Taken together, all these results demonstrated that TRIM27 was a positive oncogene for ESCC.

Growing evidences have indicated that the dysfunction of PI3/AKT signaling pathway is one of the hallmarks for human cancers [26, 27]. In the present study, our results demonstrated that the AKT inhibitor LY294002 disrupted the function of TRIM27 in ESCC cells. These results illuminated that TRIM27 was involved in PI3/AKT signaling pathway.

Previous report has demonstrated that PTEN accelerates cell apoptosis via PI3/AKT pathway [28]. Moreover, TRIM27 has enhanced the phosphorylation of AKT pathway in colorectal cancer [5]. In the present research, TRIM27 was identified to interact with PTEN and promoted its poly-ubiquitination. Therefore, our results further confirmed that TRIM27 involved in the p-AKT pathway. Moreover, TRIM27 might inhibit the apoptosis of ESCC cells via enhancing the ubiquitination of PTEN, which subsequently promoted the activity of PI3/AKT signaling pathway in ESCC cells.

Previous report has demonstrated that the glycolysis modulation is the potential anti-cancer therapy [29]. Moreover, some evidences have demonstrated that PTEN/AKT signaling enhances glycolysis in refractory acute myeloid leukemia [30]. PTEN/AKT pathway is reported as a target in suppressing glycolysis activity in cancer cells under hypoxia [31]. In this research, overexpression of TRIM27 promotes the activity of glycolysis metabolism in ESCC cells. Moreover, the protein content of GLUT1 and HKII were positively correlated with the expression of TRIM27. Moreover, the glycolysis inhibitor 3-BrPA deeply suppressed the function of TRIM27 in ESCC cells. Therefore, TRIM27 might promote the activity of glycolysis through upregulating GLUT1 and HKII in ESCC cells. It has been demonstrated that the deficiency of PTEN increases the activity of glycolysis [32]. Hence, the TRIM27/PTEN signaling pathway might also involve in the regulation of glycolysis metabolism in ESCC cells.

## Conclusion

In this study, we induced knockdown and overexpression of TRIM27 in ESCC cells. This research not only gained a deep understanding of TRIM27 but also indicated its possible target and molecule network in ESCC cells.

## Supplementary information

**Additional file 1.** Primer sequence information. **Table S1.** Human gene TRIM27 (NM_006510.4) RNAi targeting locus information. **Table S2.** The primary antibodies information.

## Data Availability

The datasets used and/or analyzed during the current study are available from the corresponding author upon reasonable request.

## Abbreviations

TRIM27: tripartite motif-containing protein 27
ESCC: esophageal squamous cell carcinoma
SNPs: single nucleotide polymorphisms
Co-IP: co-immunoprecipitation
RNAi: RNA interference
cDNA: complementary DNA
CCK-8: cell counting kit-8
IHC: immunohistochemistry
H & E: hematoxylin and eosin
IACUC: Institutional Animal Care and Use Committee
