# Supplementary material for: TRIM27 promotes the development of esophagus cancer via regulating PTEN/AKT signaling pathway
Source: Cancer Cell Int. 2019 Nov 8;19:283. doi: 10.1186/s12935-019-0998-4 (PMC6839104; doi:10.1186/s12935-019-0998-4)
Supplement: Supplementary file 1 — Additional file 1. Primer sequence information. Table S1. Human gene TRIM27 (NM_006510.4) RNAi targeting locus information. Table S2. The primary antibodies information. [file 12935_2019_998_MOESM1_ESM.pdf]

# Supplementary File1: Primer sequence information

### 1.1 Homo sapiens tripartite motif containing 27 (TRIM27), mRNA

NM_006510.4
Primer F 5' AGGACCTGCCTGACAACC 3'
Primer R 5' CTTTCCCATACCACAAAGAC 3'
Pos: 1034-1246
Amplified product: Size: 213 bps

### 1.2 Homo sapiens glyceraldehyde-3-phosphate dehydrogenase (GAPDH), transcript variant 2, mRNA

NM_001256799.2
Primer F 5' AATCCCATCACCATCTTC 3'
Primer R 5' AGGCTGTTGTCATACTTC 3'
Pos: 436-653
Amplified product: Size: 218 bps

## Supplementary Table1: Human gene TRIM27 (NM_006510.4) RNAi targeting locus information

| RNAi Targeting Locus | | Sequence |
|---|---|---|
| Name | locus positon | |
| siTRIM27-1 | 941-959 | CCCAGTTCTCTTGCAACAT |
| siTRIM27-2 | 1052-1070 | GGGCTGAAAGAATCAGGAT |
| siTRIM27-3 | 1495-1513 | GGATTCTGGGCAGTGTCTT |

## Supplementary Table 2: The primary antibodies information

| Antibody name | Source | Dilution factor |
|---|---|---|
| TRIM27 | Abcam, UK | 1:1000 |
| GLUT1 | Abcam, UK | 1:100000 |
| HK Ⅱ | Abcam, UK | 1:500 |
| Cleaved Caspase3 | CST, USA | 1:1000 |
| AKT | CST, USA | 1:1000 |
| P-AKT | CST, USA | 1:2000 |
| GAPDH | CST, USA | 1:2000 |
